# Supplementary material for: Polyketide synthase gene clusters in the ericoid mycorrhizal fungus Oidiodendron maius: genome-wide prediction, expression profiling, and functional characterization of knockout mutants
Source: Front Plant Sci. 2026 Sep 16;17:1924430. doi: 10.3389/fpls.2026.1924430 (PMC13623903; doi:10.3389/fpls.2026.1924430)
Supplement: Supplementary file 1 [file SupplementaryFile1.zip › Supplementary Materials/Supplementary Folder 1.zip!/Oidma1_AssemblyScaffolds_Repeatmasked/knownclusterblast/cluster52/jgi.p_Oidma1_184790_mibig_hits.html]

| MIBiG Protein | Description | MIBiG Cluster | MiBiG Product | % ID | % Coverage | BLAST Score | E-value |
| --- | --- | --- | --- | --- | --- | --- | --- |
| EPE34340.1 | Thiolase-like\_protein | BGC0001035 | Pneumocandin\_biosynthetic\_gene\_cluster | 42 | 101.113762928 | 1924 | 0.0 |
| OAQ83760.1 | polyketide\_synthase | BGC0001358 | Leucinostatins\_biosynthetic\_gene\_cluster | 38 | 101.829753381 | 1664 | 0.0 |
| AAD43562.2 | Fum1p | BGC0000062 | Fumonisin\_biosynthetic\_gene\_cluster | 38 | 102.704852824 | 1655 | 0.0 |
| ACB12550.1 | Fum1 | BGC0000063 | Fumonisin\_biosynthetic\_gene\_cluster | 38 | 102.784407319 | 1651 | 0.0 |
| AMY15068.1 | hexaketide\_synthase\_MF-SQHKS | BGC0001339 | Squalestatin\_S1\_biosynthetic\_gene\_cluster | 37 | 81.5035799523 | 1273 | 0.0 |
| BAJ09789.1 | polyketide\_synthase | BGC0000146 | Solanapyrone\_biosynthetic\_gene\_cluster | 33 | 106.085918854 | 1240 | 0.0 |
| AMY15057.1 | tetraketide\_synthase\_MF-SQTKS | BGC0001339 | Squalestatin\_S1\_biosynthetic\_gene\_cluster | 32 | 105.171042164 | 1184 | 0.0 |
| BAD83684.1 | PKSN\_polyketide\_synthase\_for\_alternapyrone\_biosynthesis | BGC0000012 | Alternapyrone\_biosynthetic\_gene\_cluster | 31 | 103.898170247 | 1123 | 0.0 |
| ANF07288.1 | hrPKS | BGC0001340 | Byssochlamic\_acid\_biosynthetic\_gene\_cluster | 32 | 105.568814638 | 1096 | 0.0 |
| AAB08104.3 | polyketide\_synthase | BGC0000155\_c3 | T-toxin\_biosynthetic\_gene\_cluster | 32 | 103.659506762 | 1083 | 0.0 |
| CAP95405.1 |  | BGC0001404 | Sorbicillin\_biosynthetic\_gene\_cluster | 31 | 105.369928401 | 1045 | 9.4e-305 |
| OAQ83765.1 | KR\_domain-containing\_protein | BGC0001358 | Leucinostatins\_biosynthetic\_gene\_cluster | 37 | 64.9960222753 | 977 | 2.4e-284 |
| EAA65604.1 | hypothetical\_protein | BGC0000022 | Asperfuranone\_biosynthetic\_gene\_cluster | 30 | 103.937947494 | 970 | 3.9e-282 |
| BAC20566.1 | polyketide\_synthase | BGC0000039 | Compactin\_biosynthetic\_gene\_cluster | 30 | 85.9586316627 | 826 | 1.2e-238 |
| ABA02240.1 | polyketide\_synthase | BGC0000098 | Monacolin\_K\_biosynthetic\_gene\_cluster | 30 | 85.9984089101 | 804 | 2.8e-232 |
| CBF87072.1 | polyketide\_synthase,\_putative\_(Eurofung) | BGC0001290 | Emericellamide\_A\_/\_emericellamide\_B\_biosynthetic\_gene\_cluster | 30 | 80.9466984885 | 790 | 4.2e-228 |
| AAD34559.1 | polyketide\_synthase | BGC0000089 | Lovastatin\_biosynthetic\_gene\_cluster | 30 | 85.799522673 | 787 | 6.1e-227 |
| EHA28244.1 | hypothetical\_protein | BGC0001143 | Azanigerone\_biosynthetic\_gene\_cluster | 30 | 85.3221957041 | 782 | 1.9e-225 |
| CAL69597.1 | nonribosomal\_peptide\_synthase | BGC0001049 | Tenellin\_biosynthetic\_gene\_cluster | 33 | 77.7645186953 | 769 | 1e-221 |
| AAV66110.2 | fusaridione\_A\_synthetase | BGC0000992 | Fusaridione\_A\_biosynthetic\_gene\_cluster | 32 | 82.8957836118 | 760 | 6.1e-219 |
| AKC54422.1 | fumosorinone\_biosynthesis\_polyketide\_synthase | BGC0001218 | Fumosorinone\_biosynthetic\_gene\_cluster | 32 | 76.4120922832 | 725 | 2.2e-208 |
| AGC95324.1 | CurS1 | BGC0000045 | Dehydrocurvularin\_biosynthetic\_gene\_cluster | 36 | 50.8353221957 | 665 | 3.5e-190 |
| AHV78245.1 | LasS1 | BGC0001245 | Lasiodiplodin\_biosynthetic\_gene\_cluster | 36 | 51.3922036595 | 658 | 3.2e-188 |
| EWG54266.1 | hypothetical\_protein | BGC0001190 | Fusaric\_acid\_biosynthetic\_gene\_cluster | 36 | 48.4089101034 | 647 | 5.7e-185 |
| CBX87032.1 | BcBOA9\_protein | BGC0001243\_c3 | Botryenalol\_/\_botrydial\_/\_dihydrobotrydial\_/\_botryendial\_/ | 36 | 48.7271280827 | 640 | 9.1e-183 |
| BAN19720.1 | polyketide\_synthase | BGC0001252 | ACR-toxin\_biosynthetic\_gene\_cluster | 27 | 84.9244232299 | 634 | 5e-181 |
| ACD39767.1 | reducing\_polyketide\_synthase | BGC0000077 | Hypothemycin\_biosynthetic\_gene\_cluster | 35 | 49.9204455052 | 614 | 7e-175 |
| ACD39758.1 | reducing\_polyketide\_synthase | BGC0000076 | Hypothemycin\_biosynthetic\_gene\_cluster | 35 | 49.9204455052 | 614 | 7e-175 |
| EFY94490.1 | polyketide\_synthase,\_putative | BGC0000337 | Destruxins\_biosynthetic\_gene\_cluster | 32 | 51.5513126492 | 598 | 3.1e-170 |
| BAK26562.1 | PKS-NRPS\_hybrid | BGC0000977 | Cyclopiazonic\_acid\_biosynthetic\_gene\_cluster | 34 | 48.4884645982 | 577 | 9.5e-164 |
| BAD97694.1 | Aft9-1 | BGC0000003 | AF-toxin\_biosynthetic\_gene\_cluster | 26 | 84.6857597454 | 570 | 8.9e-162 |
| EAL85129.1 | polyketide\_synthase,\_putative | BGC0001067 | Fumagillin\_biosynthetic\_gene\_cluster | 31 | 55.3301511535 | 541 | 5.8e-153 |
| CAD19091.1 | StiG\_protein | BGC0000153 | Stigmatellin\_biosynthetic\_gene\_cluster | 29 | 49.8806682578 | 489 | 2.6e-137 |
| AIT55260.1 | GulC | BGC0000072 | Gulmirecin\_biosynthetic\_gene\_cluster | 30 | 50.3977724741 | 482 | 4.2e-135 |
| EAW09117.1 | hybrid\_NRPS/PKS\_enzyme,\_putative | BGC0000983 | Cytochalasin\_biosynthetic\_gene\_cluster | 31 | 54.8130469372 | 480 | 1.2e-134 |
| AGC45623.1 | polyketide\_synthase | BGC0001394 | Phenalamide\_biosynthetic\_gene\_cluster | 32 | 46.9371519491 | 478 | 4.6e-134 |
| AAT70106.1 | CurK | BGC0001165 | Curacin\_biosynthetic\_gene\_cluster | 30 | 51.3922036595 | 478 | 6e-134 |
| AEE88279.1 | CurK | BGC0000976 | Curacin\_biosynthetic\_gene\_cluster | 30 | 51.3922036595 | 478 | 6e-134 |
| CAJ46690.1 | polyketide\_synthase | BGC0000969 | Chondramide\_biosynthetic\_gene\_cluster | 32 | 46.8178202068 | 475 | 3.9e-133 |
| EAQ86385.1 | hypothetical\_protein | BGC0001405 | Chaetoviridin\_/\_chaetomugilin\_biosynthetic\_gene\_cluster | 31 | 47.6531424025 | 475 | 3.9e-133 |
| ALD82524.1 | polyketide\_synthase | BGC0001212 | Nannocystin\_biosynthetic\_gene\_cluster | 30 | 49.8011137629 | 471 | 7.3e-132 |
| CAO91861.1 | putative\_PKS-NRPS\_protein | BGC0000968 | Chaetoglobosin\_biosynthetic\_gene\_cluster | 29 | 54.892601432 | 457 | 8.4e-128 |
| AEE88284.1 | CurF | BGC0000976 | Curacin\_biosynthetic\_gene\_cluster | 29 | 50.4375497216 | 445 | 4.3e-124 |
| ACS68554.1 | hybrid\_PKS-NRPS\_protein | BGC0001026 | NG-391\_biosynthetic\_gene\_cluster | 30 | 51.5115354018 | 444 | 7.4e-124 |
| AAT70101.1 | CurF | BGC0001165 | Curacin\_biosynthetic\_gene\_cluster | 29 | 50.4375497216 | 444 | 9.6e-124 |
| AAT28740.1 | FUSS | BGC0000064 | Fusarin\_biosynthetic\_gene\_cluster | 30 | 54.1368337311 | 442 | 2.8e-123 |
| ADN43685.1 | DmbS | BGC0001136 | Desmethylbassianin\_biosynthetic\_gene\_cluster | 29 | 55.568814638 | 439 | 3.1e-122 |
| AHA12078.1 | polyketide\_synthase\_type\_1 | BGC0001172 | Chlorizidine\_A\_biosynthetic\_gene\_cluster | 34 | 37.9474940334 | 436 | 2.6e-121 |
| CBF80487.1 | hybrid\_PKS-NRPS\_(Eurofung) | BGC0000959 | Aspyridone\_biosynthetic\_gene\_cluster | 28 | 51.8297533811 | 435 | 3.4e-121 |
| ALD82523.1 | polyketide\_synthase | BGC0001212 | Nannocystin\_biosynthetic\_gene\_cluster | 34 | 38.4645982498 | 431 | 4.9e-120 |
| AAW03325.1 | CtaB | BGC0000982 | Cystothiazole\_A\_biosynthetic\_gene\_cluster | 33 | 37.4701670644 | 431 | 6.5e-120 |
| CAE46843.1 | Type\_I\_modular\_polyketide\_synthase | BGC0000103 | Mycolactone\_biosynthetic\_gene\_cluster | 30 | 47.4144789181 | 429 | 3.2e-119 |
| CAE46851.1 | Type\_I\_modular\_polyketide\_synthase | BGC0000103 | Mycolactone\_biosynthetic\_gene\_cluster | 30 | 47.4144789181 | 429 | 3.2e-119 |
| BAJ14522.1 | polyketide\_synthase | BGC0001254 | ACT-Toxin\_II\_biosynthetic\_gene\_cluster | 29 | 52.2275258552 | 427 | 1.2e-118 |
| AFP73394.1 | FusA | BGC0001268 | Fusarin\_biosynthetic\_gene\_cluster | 30 | 47.4940334129 | 425 | 4.6e-118 |
| AAY28226.1 | HbmAII | BGC0000074 | Herbimycin\_biosynthetic\_gene\_cluster | 29 | 50.4375497216 | 419 | 2.5e-116 |
| AEE88281.1 | CurI | BGC0000976 | Curacin\_biosynthetic\_gene\_cluster | 32 | 38.3054892601 | 418 | 7.4e-116 |
| AAT70104.1 | CurI | BGC0001165 | Curacin\_biosynthetic\_gene\_cluster | 32 | 38.3054892601 | 418 | 7.4e-116 |
| BAB69198.1 | modular\_polyketide\_synthase | BGC0000117 | Oligomycin\_biosynthetic\_gene\_cluster | 28 | 47.2951471758 | 415 | 4.8e-115 |
| CAE46850.1 | Type\_I\_modular\_polyketide\_synthase | BGC0000103 | Mycolactone\_biosynthetic\_gene\_cluster | 28 | 47.6531424025 | 414 | 8.2e-115 |
| AAO06917.1 | GdmAII | BGC0000066 | Geldanamycin\_biosynthetic\_gene\_cluster | 29 | 50.7159904535 | 411 | 5.3e-114 |
| AFV96138.1 | polyketide\_synthase | BGC0001064 | Cylindrocyclophane\_biosynthetic\_gene\_cluster | 33 | 37.4701670644 | 411 | 9e-114 |
| AAT70107.1 | CurL | BGC0001165 | Curacin\_biosynthetic\_gene\_cluster | 32 | 37.8679395386 | 410 | 1.5e-113 |
| BAK64650.1 | polyketide\_synthase | BGC0000135 | Reveromycin\_biosynthetic\_gene\_cluster | 29 | 46.9769291965 | 410 | 1.5e-113 |
| AEE88278.1 | CurL | BGC0000976 | Curacin\_biosynthetic\_gene\_cluster | 32 | 37.8679395386 | 410 | 1.5e-113 |
| BAK64637.1 | polyketide\_synthase | BGC0000135 | Reveromycin\_biosynthetic\_gene\_cluster | 28 | 46.5791567224 | 405 | 3.8e-112 |
| AGO86662.1 | equisetin\_synthetase | BGC0001255 | Equisetin\_biosynthetic\_gene\_cluster | 29 | 52.7446300716 | 405 | 5e-112 |
| AAY28225.1 | HbmAI | BGC0000074 | Herbimycin\_biosynthetic\_gene\_cluster | 28 | 47.5735879077 | 402 | 4.2e-111 |
| ACV42478.1 | polyketide\_synthase | BGC0000043 | Curacin\_A\_biosynthetic\_gene\_cluster | 32 | 38.5043754972 | 401 | 5.5e-111 |
| AAT70108.1 | CurM | BGC0001165 | Curacin\_biosynthetic\_gene\_cluster | 32 | 38.5043754972 | 401 | 5.5e-111 |
| AEE88277.1 | CurM | BGC0000976 | Curacin\_biosynthetic\_gene\_cluster | 32 | 38.5043754972 | 401 | 5.5e-111 |
| AAS98782.1 | JamK | BGC0001001 | Jamaicamide\_biosynthetic\_gene\_cluster | 31 | 39.3794749403 | 400 | 1.6e-110 |
| ACZ57548.1 | polyketide\_synthase | BGC0000046 | Depudecin\_biosynthetic\_gene\_cluster | 33 | 33.492442323 | 393 | 2.5e-108 |
| BAQ21940.1 | putative\_Type\_I\_polyketide\_synthase | BGC0001204 | Versipelostatin\_biosynthetic\_gene\_cluster | 28 | 47.4144789181 | 393 | 2.5e-108 |
| BAQ21947.1 | putative\_type\_I\_polyketide\_synthase | BGC0001204 | Versipelostatin\_biosynthetic\_gene\_cluster | 27 | 47.6133651551 | 392 | 3.3e-108 |
| BAQ25512.1 | type\_I\_polyketide\_synthase | BGC0001288 | Maklamicin\_biosynthetic\_gene\_cluster | 28 | 47.7724741448 | 389 | 2.2e-107 |
| AIA58899.1 | HRPKS | BGC0001141 | Brefeldin\_biosynthetic\_gene\_cluster | 33 | 32.0206841687 | 383 | 2.6e-105 |
| AAK57187.1 | MxaC | BGC0001022 | Myxalamid\_biosynthetic\_gene\_cluster | 28 | 50.0 | 378 | 5e-104 |
| EAU29808.1 | hypothetical\_protein | BGC0001400 | Citreoviridin\_biosynthetic\_gene\_cluster | 27 | 54.5743834527 | 373 | 2.7e-102 |
| AEC13069.1 | fosC | BGC0000060 | Fostriecin\_biosynthetic\_gene\_cluster | 31 | 37.5099443119 | 369 | 2.3e-101 |
| CAQ34929.1 | putative\_polyketide\_synthase | BGC0000986 | Dkxanthene\_biosynthetic\_gene\_cluster | 31 | 37.8281622912 | 361 | 8.2e-99 |
| ABV83229.1 | CppB | BGC0000116 | Nystatin-like\_Pseudonocardia\_polyene\_biosynthetic\_gene\_cluster | 31 | 38.0270485282 | 361 | 1.1e-98 |
| BAG84248.1 | putative\_polyketide\_synthase | BGC0000257 | Prodigiosin\_biosynthetic\_gene\_cluster | 31 | 38.2259347653 | 354 | 7.7e-97 |
| AAO65798.1 | monensin\_polyketide\_synthase\_modules\_3\_and\_4 | BGC0000100 | Monensin\_biosynthetic\_gene\_cluster | 29 | 41.5274463007 | 351 | 8.5e-96 |
| BAC20564.1 | polyketide\_synthase | BGC0000039 | Compactin\_biosynthetic\_gene\_cluster | 27 | 53.0628480509 | 350 | 1.9e-95 |
| KGO40478.1 | Acyl\_transferase/acyl\_hydrolase/lysophospholipase | BGC0001205 | Communesin\_biosynthetic\_gene\_cluster | 33 | 31.0262529833 | 347 | 1.2e-94 |
| AFV96142.1 | polyketide\_synthase | BGC0001064 | Cylindrocyclophane\_biosynthetic\_gene\_cluster | 30 | 37.3906125696 | 339 | 2.6e-92 |
| BI06\_RS39060 | polyketide\_synthase | BGC0001232 | Leucanicidin\_biosynthetic\_gene\_cluster | 29 | 38.4645982498 | 338 | 7.4e-92 |
| CUI25702.1 | Tu3010\_Hybrid\_PKS-NRPS\_system | BGC0001352 | Thiotetronate\_Tu\_3010\_biosynthetic\_gene\_cluster | 27 | 47.215592681 | 323 | 1.9e-87 |
| ALJ49921.1 | TtmH | BGC0001236 | Thiotetroamide\_biosynthetic\_gene\_cluster | 29 | 37.5099443119 | 318 | 6.1e-86 |
| CUI25744.1 | Polyketide\_synthase/Nonribosomal\_peptide\_synthase | BGC0001354 | U-68204\_biosynthetic\_gene\_cluster | 29 | 37.8679395386 | 315 | 5.2e-85 |
| AAP33839.2 | ochratoxin\_A\_polyketide\_synthase | BGC0001030 | Ochratoxin\_A\_biosynthetic\_gene\_cluster | 31 | 31.4638027049 | 312 | 4.4e-84 |
| AAS98783.1 | JamL | BGC0001001 | Jamaicamide\_biosynthetic\_gene\_cluster | 38 | 21.5990453461 | 298 | 6.5e-80 |
| ABM21569.1 | CrpA | BGC0000975 | Cryptophycin\_biosynthetic\_gene\_cluster | 26 | 48.7271280827 | 298 | 8.5e-80 |
| ADM79459.1 | PKS16\_protein | BGC0001266 | Grayanic\_acid\_biosynthetic\_gene\_cluster | 28 | 38.4248210024 | 291 | 1e-77 |
| XP\_001220460.1 | hypothetical\_protein | BGC0001182 | Chaetoglobosins\_biosynthetic\_gene\_cluster | 31 | 31.8217979316 | 285 | 5.7e-76 |
| AEU11005.1 | NpnA | BGC0001029 | Nostophycin\_biosynthetic\_gene\_cluster | 31 | 27.923627685 | 277 | 2e-73 |
| ABB76806.1 | polyketide\_synthase\_2 | BGC0000155\_c2 | T-toxin\_biosynthetic\_gene\_cluster | 30 | 29.6738265712 | 275 | 5.9e-73 |
| CAQ18832.1 | polyketide\_synthase | BGC0000954 | Ajudazol\_biosynthetic\_gene\_cluster | 32 | 24.6221161496 | 273 | 2.9e-72 |
| CAL69889.1 | RhiB\_protein | BGC0001112 | Rhizoxins\_biosynthetic\_gene\_cluster | 31 | 26.6905330151 | 257 | 1.7e-67 |
| AAQ82567.1 | FscE | BGC0000034 | Candicidin\_biosynthetic\_gene\_cluster | 26 | 31.7422434368 | 231 | 1.3e-59 |
| AAT70103.1 | CurH | BGC0001165 | Curacin\_biosynthetic\_gene\_cluster | 31 | 21.2808273667 | 230 | 2.9e-59 |
| AEE88282.1 | CurH | BGC0000976 | Curacin\_biosynthetic\_gene\_cluster | 31 | 21.2808273667 | 230 | 2.9e-59 |
| CUW01174.1 | Type\_I\_polyketide\_synthase | BGC0001359 | PM100117\_/\_PM100118\_biosynthetic\_gene\_cluster | 28 | 31.7422434368 | 227 | 1.8e-58 |
| AHE80994.1 | PieA4 | BGC0001169 | Piericidin\_A1\_biosynthetic\_gene\_cluster | 27 | 31.4638027049 | 223 | 2.7e-57 |
| AAD03047.1 | type\_I\_polyketide\_synthase | BGC0000041 | Coronafacic\_acid\_biosynthetic\_gene\_cluster | 27 | 29.753381066 | 221 | 1.3e-56 |
| CAP95404.1 |  | BGC0001404 | Sorbicillin\_biosynthetic\_gene\_cluster | 30 | 21.3206046142 | 220 | 1.7e-56 |
| AEZ54377.1 | PieA4 | BGC0000124 | Piericidin\_A1\_biosynthetic\_gene\_cluster | 29 | 27.6451869531 | 218 | 8.6e-56 |
| AAZ77694.1 | ChlA2 | BGC0000036 | Chlorothricin\_biosynthetic\_gene\_cluster | 26 | 32.6968973747 | 218 | 8.6e-56 |
| CCP20048.1 | divL1\_protein | BGC0001119 | Divergolide\_biosynthetic\_gene\_cluster | 30 | 20.92283214 | 218 | 1.1e-55 |
| AAO65797.1 | monensin\_polyketide\_synthase\_module\_2 | BGC0000100 | Monensin\_biosynthetic\_gene\_cluster | 29 | 24.860779634 | 217 | 1.9e-55 |
| BAD08358.1 | polyketide\_synthase\_modules\_4 | BGC0000167 | Vicenistatin\_biosynthetic\_gene\_cluster | 27 | 32.2991249006 | 217 | 2.5e-55 |
| ACN69991.1 | polyketide\_synthase | BGC0000079 | Indanomycin\_biosynthetic\_gene\_cluster | 27 | 29.1567223548 | 216 | 4.3e-55 |
| AEU17897.1 | putative\_type\_I\_PKS | BGC0001072 | Simocyclinone\_biosynthetic\_gene\_cluster | 30 | 22.5139220366 | 216 | 4.3e-55 |
| AAZ77698.1 | ChlA5 | BGC0000036 | Chlorothricin\_biosynthetic\_gene\_cluster | 29 | 24.0652346858 | 216 | 4.3e-55 |
| AHH99926.1 | PKS\_I | BGC0000002 | Aculeximycin\_biosynthetic\_gene\_cluster | 27 | 29.1964996022 | 215 | 9.5e-55 |
| CAO98852.1 | polyketide\_synthase\_AufI | BGC0000023 | Aurafuron\_biosynthetic\_gene\_cluster | 27 | 26.3325377884 | 214 | 1.6e-54 |
| AAF71767.1 | NysJ | BGC0000115 | Nystatin\_biosynthetic\_gene\_cluster | 29 | 26.9291964996 | 214 | 1.6e-54 |
| ABY21540.1 | AngAIII | BGC0000018 | Angolamycin\_biosynthetic\_gene\_cluster | 28 | 27.6054097056 | 213 | 3.6e-54 |
| ACY06288.1 | type\_I\_polyketide\_synthase | BGC0001042 | Sanglifehrin\_A\_biosynthetic\_gene\_cluster | 26 | 32.6571201273 | 212 | 6.2e-54 |
| AEZ54374.1 | PieA1 | BGC0000124 | Piericidin\_A1\_biosynthetic\_gene\_cluster | 28 | 24.1050119332 | 211 | 1e-53 |
| AGY62754.1 | EbeB | BGC0000051 | Ebelactone\_biosynthetic\_gene\_cluster | 26 | 32.8162291169 | 210 | 1.8e-53 |
| AJO72742.1 | Type\_I\_modular\_polyketide\_synthase | BGC0001381 | Brasilinolide\_biosynthetic\_gene\_cluster | 28 | 29.5147175815 | 210 | 1.8e-53 |
| ADB23403.1 | polyketide\_synthase\_type\_I | BGC0001062 | TLN-05220\_biosynthetic\_gene\_cluster | 26 | 33.4526650756 | 210 | 1.8e-53 |
| AFV30248.1 | polyketide\_synthase | BGC0000075 | Hygrocin\_biosynthetic\_gene\_cluster | 26 | 31.8217979316 | 210 | 2.3e-53 |
| AEZ53952.1 | polyketide\_synthase | BGC0000144 | Salinomycin\_biosynthetic\_gene\_cluster | 29 | 22.5934765314 | 203 | 2.9e-51 |
| AAG23265.1 | polyketide\_synthase\_extender\_module\_2 | BGC0000148 | Spinosad\_biosynthetic\_gene\_cluster | 29 | 27.6451869531 | 203 | 2.9e-51 |
| CAE45671.1 | borrelidin\_polyketide\_synthase,\_type\_I | BGC0000031 | Borrelidin\_biosynthetic\_gene\_cluster | 28 | 22.0365950676 | 201 | 1.1e-50 |
| BAC68128.1 | modular\_polyketide\_synthase | BGC0000059 | Filipin\_biosynthetic\_gene\_cluster | 29 | 23.8663484487 | 201 | 1.4e-50 |
| AAO06916.1 | GdmAI | BGC0000066 | Geldanamycin\_biosynthetic\_gene\_cluster | 27 | 29.6340493238 | 200 | 1.9e-50 |
| ABB86408.1 | GelA | BGC0000067 | Geldanamycin\_biosynthetic\_gene\_cluster | 27 | 29.6340493238 | 200 | 1.9e-50 |
| AAP42856.1 | NanA2 | BGC0000105 | Nanchangmycin\_biosynthetic\_gene\_cluster | 27 | 29.554494829 | 199 | 4.1e-50 |
| AAG23262.1 | polyketide\_synthase\_extender\_modules\_8-10 | BGC0000148 | Spinosad\_biosynthetic\_gene\_cluster | 31 | 24.3038981702 | 199 | 5.4e-50 |
| AHH99919.1 | PKS\_I | BGC0000002 | Aculeximycin\_biosynthetic\_gene\_cluster | 27 | 24.6221161496 | 195 | 7.8e-49 |
| AAP42859.1 | NanA5 | BGC0000105 | Nanchangmycin\_biosynthetic\_gene\_cluster | 28 | 24.860779634 | 195 | 7.8e-49 |
| ACO94499.1 | polyketide\_synthase\_type\_I | BGC0000097 | ML-449\_biosynthetic\_gene\_cluster | 26 | 25.8949880668 | 190 | 1.9e-47 |
| AAC46026.1 | polyketide\_synthase\_modules\_4\_and\_5 | BGC0000113 | Niddamycin\_biosynthetic\_gene\_cluster | 25 | 32.338902148 | 190 | 2.5e-47 |
| ABC84457.1 | NigAII | BGC0000114 | Nigericin\_biosynthetic\_gene\_cluster | 28 | 24.5823389021 | 190 | 2.5e-47 |
| ADX66459.1 | ScnS4 | BGC0000108 | Natamycin\_biosynthetic\_gene\_cluster | 27 | 25.8552108194 | 189 | 4.3e-47 |
| AAC01714.1 | RifE | BGC0000136 | Rifamycin\_biosynthetic\_gene\_cluster | 26 | 24.3436754177 | 188 | 1.2e-46 |
| ACO94471.1 | polyketide\_synthase\_type\_I | BGC0000029 | BE-14106\_biosynthetic\_gene\_cluster | 27 | 26.0143198091 | 186 | 3.6e-46 |
| ctg1\_orf23 |  | BGC0001013 | Meridamycin\_biosynthetic\_gene\_cluster | 26 | 32.4184566428 | 186 | 4.7e-46 |
| ABC87512.1 | polyketide\_synthase | BGC0001011 | Meridamycin\_biosynthetic\_gene\_cluster | 26 | 32.4184566428 | 186 | 4.7e-46 |
| BAB69199.1 | modular\_polyketide\_synthase | BGC0000117 | Oligomycin\_biosynthetic\_gene\_cluster | 24 | 27.1678599841 | 184 | 1.4e-45 |
| ACY06286.1 | polyketide\_synthase | BGC0001042 | Sanglifehrin\_A\_biosynthetic\_gene\_cluster | 25 | 31.8217979316 | 183 | 3.1e-45 |
| BAH02270.1 | polyketide\_synthase | BGC0000126 | Pladienolide\_biosynthetic\_gene\_cluster | 26 | 28.9976133652 | 182 | 5.2e-45 |
| CAD19087.1 | StiC\_protein | BGC0000153 | Stigmatellin\_biosynthetic\_gene\_cluster | 26 | 27.4860779634 | 182 | 5.2e-45 |
| ADM46357.1 | polyketide\_synthase | BGC0000106 | Naphthomycin\_biosynthetic\_gene\_cluster | 27 | 27.0883054893 | 181 | 8.9e-45 |
| ADM46358.1 | polyketide\_synthase | BGC0000106 | Naphthomycin\_biosynthetic\_gene\_cluster | 26 | 27.6451869531 | 181 | 8.9e-45 |
| ADM46359.1 | polyketide\_synthase | BGC0000106 | Naphthomycin\_biosynthetic\_gene\_cluster | 27 | 28.6396181384 | 181 | 1.2e-44 |
| ADM46360.1 | polyketide\_synthase | BGC0000106 | Naphthomycin\_biosynthetic\_gene\_cluster | 28 | 23.8663484487 | 180 | 2e-44 |
| CAC20919.1 | PimS4\_protein | BGC0000125 | Pimaricin\_biosynthetic\_gene\_cluster | 27 | 27.4860779634 | 178 | 7.5e-44 |
| ABB88521.1 | polyketide\_synthase\_type\_I | BGC0000050 | E-837\_biosynthetic\_gene\_cluster | 27 | 23.6674622116 | 178 | 7.5e-44 |
| WP\_063764078.1 | polyketide\_synthase | BGC0001348 | JBIR-100\_biosynthetic\_gene\_cluster | 26 | 27.6451869531 | 177 | 2.2e-43 |
| ACO94488.1 | polyketide\_synthase\_type\_I | BGC0000097 | ML-449\_biosynthetic\_gene\_cluster | 28 | 23.3094669849 | 176 | 2.9e-43 |
| AKD43753.1 | HerB | BGC0001349 | Heronamide\_biosynthetic\_gene\_cluster | 28 | 23.9061256961 | 176 | 3.7e-43 |
| AKD43764.1 | HerF | BGC0001349 | Heronamide\_biosynthetic\_gene\_cluster | 28 | 23.2299124901 | 176 | 3.7e-43 |
| ACO94460.1 | polyketide\_synthase\_type\_I | BGC0000029 | BE-14106\_biosynthetic\_gene\_cluster | 27 | 23.9061256961 | 174 | 1.4e-42 |
| ADC79618.1 | BafAIII | BGC0000028 | Bafilomycin\_biosynthetic\_gene\_cluster | 29 | 20.0875099443 | 173 | 3.2e-42 |
| BAC57032.1 | protomycinolide\_IV\_synthase\_5 | BGC0000102 | Mycinamicin\_biosynthetic\_gene\_cluster | 28 | 26.4120922832 | 170 | 2.1e-41 |
| AAZ94391.1 | modular\_polyketide\_synthase | BGC0000040 | Concanamycin\_A\_biosynthetic\_gene\_cluster | 26 | 30.0318217979 | 169 | 4.6e-41 |
| BAF92601.1 | iterative\_type\_I\_PKS | BGC0000118 | Pactamycin\_biosynthetic\_gene\_cluster | 29 | 21.400159109 | 169 | 6e-41 |
| ACJ24875.1 | 6-methylsalicylic\_acid\_synthase | BGC0000119 | Pactamycin\_biosynthetic\_gene\_cluster | 29 | 21.400159109 | 169 | 6e-41 |
| BAG85027.1 | putative\_polyketide\_synthase | BGC0000086 | Lasalocid\_biosynthetic\_gene\_cluster | 27 | 24.3038981702 | 168 | 1e-40 |
| AEZ64503.1 | Herd | BGC0001065 | Herboxidiene\_biosynthetic\_gene\_cluster | 28 | 24.2641209228 | 168 | 1e-40 |
| CAQ64687.1 | lasalocid\_modular\_polyketide\_synthase | BGC0000087 | Lasalocid\_biosynthetic\_gene\_cluster | 27 | 24.3038981702 | 168 | 1e-40 |
| AHB82062.1 | polyketide\_synthase | BGC0001231 | Microsclerodermins\_biosynthetic\_gene\_cluster | 25 | 31.9809069212 | 166 | 3.9e-40 |
| ACB46487.1 | polyketide\_synthase | BGC0000082 | Kijanimicin\_biosynthetic\_gene\_cluster | 28 | 23.7867939539 | 164 | 1.5e-39 |
| AHE80995.1 | PieA5 | BGC0001169 | Piericidin\_A1\_biosynthetic\_gene\_cluster | 26 | 24.661893397 | 161 | 9.5e-39 |
| BAP34739.1 | type\_I\_polyketide\_synthase | BGC0000078 | Incednine\_biosynthetic\_gene\_cluster | 27 | 23.2696897375 | 161 | 1.2e-38 |
| BAP34734.1 | type\_I\_polyketide\_synthase | BGC0000078 | Incednine\_biosynthetic\_gene\_cluster | 28 | 21.5990453461 | 158 | 1.1e-37 |
| ABB05104.1 | LipPks3 | BGC0001003 | Lipomycin\_biosynthetic\_gene\_cluster | 26 | 25.815433572 | 153 | 2.6e-36 |
| BAP34733.1 | type\_I\_polyketide\_synthase | BGC0000078 | Incednine\_biosynthetic\_gene\_cluster | 27 | 23.0708035004 | 149 | 4.9e-35 |
| CAJ88187.2 | putative\_type\_I\_modular\_polyketide\_synthase | BGC0000151 | Stambomycin\_biosynthetic\_gene\_cluster | 26 | 23.707239459 | 146 | 4.1e-34 |
